# Supplementary material for: Retinal oxygen supply shaped the functional evolution of the vertebrate eye
Source: eLife. 2019 Dec 10;8:e52153. doi: 10.7554/eLife.52153 (PMC6904217; doi:10.7554/eLife.52153)
Supplement: Supplementary file 1. — Most data points were generated within this study, but literature data on maximal retinal thickness and Root effect magnitude as well as literature observations on different capillary types was included in the analysis. [file elife-52153-supp1.docx]

|  | **This study** | | | **Literature** | | | **Total** | | |
| --- | --- | --- | --- | --- | --- | --- | --- | --- | --- |
|  | Ray-finned fishes | Other vertebrates | All vertebrates | Ray-finned fishes | Other vertebrates | All vertebrates | Ray-finned fishes | Other vertebrates | All vertebrates |
| Root effect magnitude | 40 | 3 | 43 | 29 | 7 | 36 | 69 | 10 | 79 |
| Maximal retinal thickness | 34 | 5 | 39 | 0 | 14 | 14 | 34 | 19 | 53 |
| Retinal layer thicknesses | 31 | 6 | 37 | 0 | 0 | 0 | 31 | 6 | 37 |
| Choroid *rete mirabile* surface area | 19 | 0 | 19 | 0 | 0 | 0 | 19 | 0 | 19 |
| Magnitude of pre-retinal capillarization | 18 | 1 | 19 | 0 | 0 | 0 | 18 | 1 | 19 |
| Presence of choroid *rete mirabile* | 44 | 8 | 52 | 256 | 87 | 343 | 300 | 95 | 395 |
| Presence of intra-retinal capillarization | 34 | 8 | 42 | 2 | 49 | 51 | 36 | 57 | 93 |
| Presence of pre-retinal capillarization | 32 | 8 | 40 | 0 | 3 | 3 | 32 | 11 | 43 |
| Eye mass | 75 | 4 | 79 | 0 | 0 | 0 | 75 | 4 | 79 |
| **Total** | **79** | **8** | **87** | **260** | **89** | **349** | **309** | **95** | **404** |
