# Supplementary material for: Retinal oxygen supply shaped the functional evolution of the vertebrate eye
Source: eLife. 2019 Dec 10;8:e52153. doi: 10.7554/eLife.52153 (PMC6904217; doi:10.7554/eLife.52153)
Supplement: Supplementary file 2. — CT, computed tomography; n, numbers of replicates for ultrasound; NA, not available; US, ultrasound. Values are means ± standard deviations. [file elife-52153-supp2.docx]

| Species name | Body mass  (g) | Length  (cm) | n | [Benzocaine]  (mg/L) | US transducer frequency  (MHz) | Micro-CT resolution  (µm pixel^-1^) |
| --- | --- | --- | --- | --- | --- | --- |
| *Ambystoma mexicanum* | 56.7 ± 13.7 | 18.7 ± 1.62 | 3 | 0.20 | 48 | 6 |
| *Lepidosiren paradoxa* | 77.8 ± 34.9 | 27.3 ± 4.26 | 3 | 0.15 | 48 | 3.5 |
| *Protopterus annectens* | 1726 | 94 | 1 | 0.20 | 40 | 6 |
| *Polypterus senegalus* | 9.11 ± 0.305 | 11.9 ± 0.327 | 3 | 0.11 | 48 | 3.5 |
| *Acipenser baerii* | 1980 ± 533 | 71.6 ± 7.41 | 3 | 0.15 | 40 | 10 |
| *Lepisosteus oculatus* | 2.81 ± 0.662 | 11.1 ± 0.941 | 3 | 0.10 | 48 | 3.5 |
| *Anguilla anguilla* | 727 ± 248 | 65.8 ± 9.83 | 4 | 0.15 | 40 | 3.5 |
| *Pantodon buchholzi* | 4.30 ± 0.180 | 8.60 ± 0.283 | 3 | 0.15 | 48 | 3.5 |
| *Gnathonemus petersii* | 14.1 ± 2.52 | 12.9 ± 0.589 | 3 | 0.11 | 48 | 3.5 |
| *Chitala ornata* | 143 ± 18.6 | 26.5 ± 0.591 | 3 | 0.15 | 40 | 6 |
| *Carassius auratus* | 339 ± 111 | 26.0 ± 2.92 | 4 | 0.08 | 40 | 10 |
| *Pangio kuhlii* | 0.390 ± 0.0374 | 6.40 ± 0.712 | 3 | 0.11 | 48 | 3.5 |
| *Pygocentrus nattereri* | 191 ± 106 | 18.9 ± 3.10 | 5 | 0.08 | 40 | 10 |
| *Astyanax mexicanus* (Surface) | 4.07 ± 2.318 | 7.11 ± 0.915 | 4 | 0.06 | 48 | 6 |
| *Astyanax mexicanus* (Pachón) | 2.94 ± 1.409 | 5.82 ± 0.691 | 4 | 0.09 | 48 | 6 |
| *Astyanax mexicanus* (Micos) | 2.78 ± 0.516 | 6.08 ± 0.59 | 4 | 0.06 | 48 | 6 |
| *Astyanax mexicanus* (Chica) | 0.75 ± 0.107 | 4.1 ± 0.216 | 3 | 0.09 | 48 | 6 |
| *Apteronotus albifrons* | 6.91 ± 3.32 | 10.9 ± 1.72 | 3 | 0.06 | 48 | 3.5 |
| *Pangasianodon hypophthalmus* | 602 ± 300 | 33.9 ± 5.14 | 6 | 0.15 | 40 | 3.5 |
| *Clarias batrachus* | 36.0 ± 5.76 | 16.9 ± 0.983 | 4 | 0.15 | 48 | 3.5 |
| *Oncorhynchus mykiss* | 448 ± 116 | 29.3 ± 2.86 | 4 | 0.05 | 40 | 10 |
| *Gadus morhua* | 236 ± 61.0 | 32.3 ± 2.75 | 2 | 0.05 | 40 | 10 |
| *Ctenolabrus rupestris* | 27.7 ± 6.73 | 12.5 ± 0.86 | 4 | 0.06 | 40 | 6 |
| *Dicentrarchus labrax* | 1067 ± 167 | 44.5 ± 2.97 | 6 | 0.05 | 21 | 15 |
| *Gasterosteus aculeatus* | 2.19 ± 0.63 | 6.18 ± 0.51 | 6 | 0.06 | 48 | 3.5 |
| *Perca fluviatilis* | 394 ± 79.7 | 28.5 ± 1.50 | 4 | 0.05 | 40 | 10 |
| *Pterophyllum scalare* | NA | 8.13 ± 0.65 | 3 | 0.08 | 40 | 6 |
| *Pleuronectes platessa* | 379 ± 8.0 | 33.2 ± 0.15 | 2 | 0.06 | 40 | 10 |
| *Parachanna obscura* | 25.1 ± 9.25 | 14.1 ± 1.98 | 3 | 0.38 | 40 | 6 |
| *Mastacembelus erythrotaenia* | 15.7 ± 2.33 | 20.9 ± 0.785 | 4 | 0.23 | 48 | 6 |
| *Monopterus albus* | 270 ± 23.0 | 54.2 ± 2.11 | 6 | 0.30 | 48 | 3.5 |
