# Supplementary material for: Retinal oxygen supply shaped the functional evolution of the vertebrate eye
Source: eLife. 2019 Dec 10;8:e52153. doi: 10.7554/eLife.52153 (PMC6904217; doi:10.7554/eLife.52153)
Supplement: Supplementary file 3. — The coefficient of variance (CV) was calculated as the ratio between standard deviation and sample mean. Values are percentages. CRM, choroid rete mirabile; n, number of replicates for ultrasound CV analysis; T, thickness; V, Volume. [file elife-52153-supp3.docx]

| Species | *T*(Retina) | *V*(retina) | *V*(CRM) | *V*(eye) | n |
| --- | --- | --- | --- | --- | --- |
| *Ambystoma mexicanum* | 16.4 | 8.05 | NA | 8.51 | 3 |
| *Lepidosiren paradoxa* | 2.54 | 14.1 | NA | 23.7 | 3 |
| *Protopterus annectens* | 0 | 0 | NA | 0 | 1 |
| *Polypterus senegalensis* | 2.18 | 6.82 | NA | 11.6 | 3 |
| *Acipenser baerii* | 5.46 | 9.1 | NA | 7.59 | 3 |
| *Lepisosteus oculatus* | 9.68 | 15.6 | NA | 14.4 | 3 |
| *Anguilla anguilla* | 1.64 | 14 | NA | 17.3 | 3 |
| *Pantadon bucholzi* | 3.26 | 7.27 | 31.3 | 3.97 | 3 |
| *Chitala ornata* | 0.919 | 2.33 | 19 | 5.28 | 3 |
| *Gnathonemus petersii* | 9.35 | 25.9 | NA | 24 | 3 |
| *Carassius auratus* | 5.75 | 15.4 | 6.41 | 16.6 | 3 |
| *Pangio kuhlii* | 5.12 | 19.3 | NA | 38.3 | 3 |
| *Pygocentrus nattereri* | 5.94 | 51.7 | 11.8 | 68.3 | 5 |
| *Astyanax mexicanus (Surface)* | 2.99 | 14.9 | 4.95 | 13.1 | 3 |
| *Astyanax mexicanus (Micos)* | 4.78 | 12.4 | 12.5 | 19.3 | 3 |
| *Apteronotus albifrons* | 1.46 | 14.4 | NA | 19.3 | 3 |
| *Pangasianodon hypophthalmus* | 5.37 | 16.1 | NA | 22.2 | 3 |
| *Clarias batrachus* | 6.55 | 10.9 | NA | 7.91 | 3 |
| *Oncorhynchus mykiss* | 9.92 | 10.2 | 13.3 | 14.7 | 3 |
| *Gadus morhua* | 0.81 | 26 | 8.44 | 26.9 | 2 |
| *Ctenolabrus rupestris* | 10.1 | 6.36 | 12.2 | 9.17 | 3 |
| *Dicentrarchus labrax* | 4.29 | 11.3 | 5.18 | 33.7 | 3 |
| *Gasterosteus aculeatus* | 6.37 | 17.7 | 12.9 | 22.9 | 3 |
| *Perca fluviatilis* | 11.5 | 9.27 | 5.64 | 7.94 | 3 |
| *Pterophyllum scalare* | 3.22 | 6.87 | 11.7 | 17.2 | 3 |
| *Pleuronectes platessa* | 5.61 | 7.69 | 13.5 | 11.2 | 2 |
| *Channa obscura* | 6.31 | 11.3 | 6.99 | 14.5 | 3 |
| *Mastacembelus eryhrotania* | 8.26 | 7.82 | 8.48 | 4.93 | 3 |
| *Monopterus albus* | 3.29 | 15.9 | NA | 3.96 | 3 |
