# Supplementary figures and images for: Retinal oxygen supply shaped the functional evolution of the vertebrate eye
Source: eLife. 2019 Dec 10;8:e52153. doi: 10.7554/eLife.52153 (PMC6904217; doi:10.7554/eLife.52153)

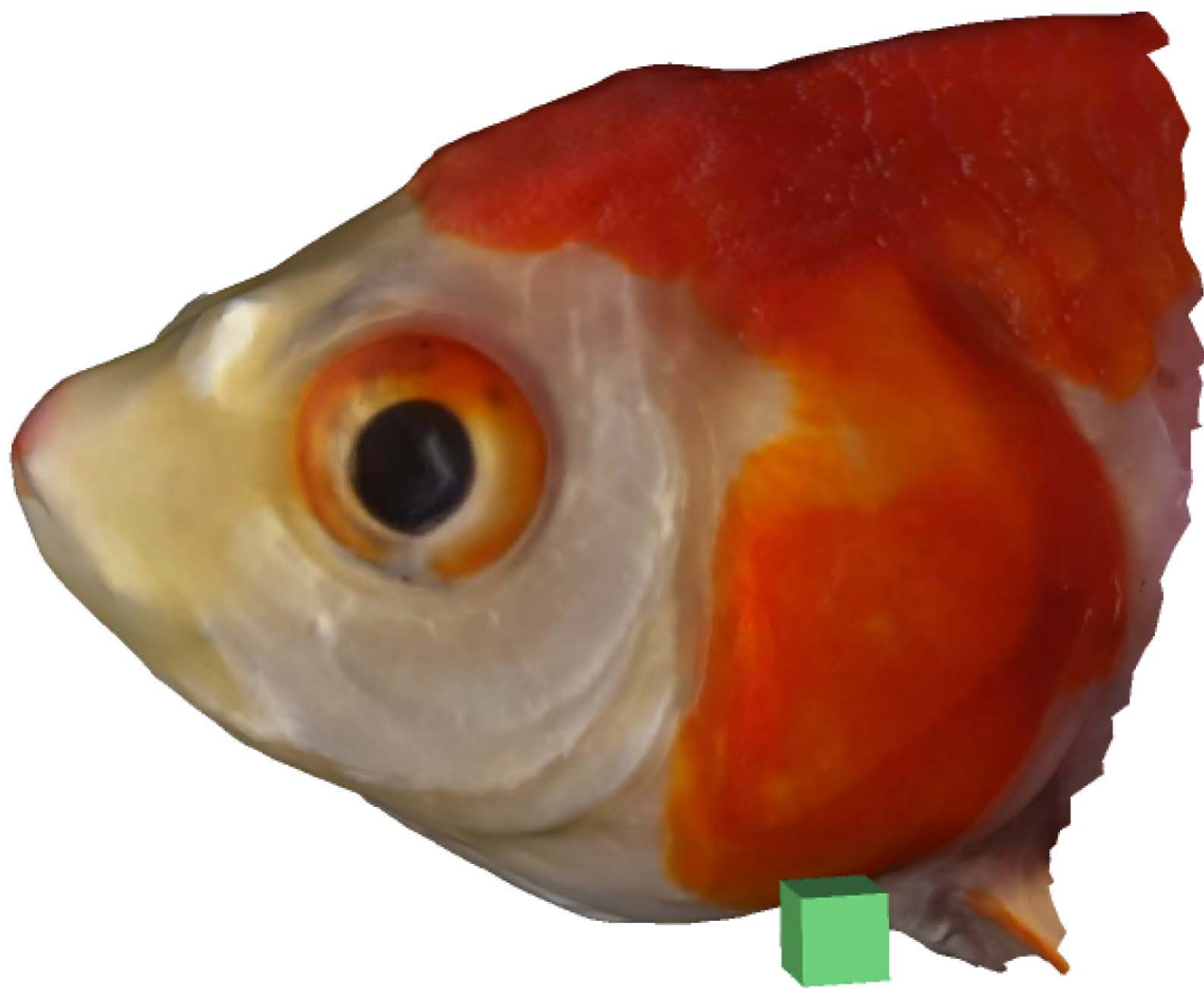

Supplement: Supplementary file 4. — Three-dimensionally rendered interactive model generated from micro-computed tomography imaging of a goldfish after an arterial injection of a BaSO4-based contrast agent. The interactive file should be viewed in Adobe Acrobat Readernineor higher. To activate the 3D feature, click the model. Using the cursor, it is possible to rotate, zoom, and pan the model. All segments of the model can be turned on/off or made transparent. The model tree is a hierarchy containing several sublayers that can be opened (+). Pre-defined views similar to Figure 3—figure supplement 1A and C can be selected below the model tree [file elife-52153-supp4.pdf]
